# Nanofluidic proton channels based on a 2D layered glass membrane with improved aqueous and acid stability†

**DOI:** 10.1039/d2ra03848j

**Published:** 2022-10-17

**Authors:** Kuiguang Luo, Tao Huang, Qi Li, Junchao Lao, Jun Gao, Yi Tang

**Affiliations:** School of Physics and Optoelectronics, Xiangtan University Xiangtan 411105 P. R. China tangyii@163.com; Qingdao Institute of Bioenergy and Bioprocess Technology Chinese Academy of Sciences Qingdao 266101 P. R. China; Shandong Energy Institute Qingdao 266101 P. R. China; School of Materials Science and Engineering, Institute of Marine Biobased Materials, Qingdao University Qingdao 266071 P. R. China; School of Chemical Engineering, Sichuan University Chengdu 610065 P. R. China; Shanghai Key Lab of Advanced High-Temperature Materials and Precision Forming, School of Materials Science and Engineering, Shanghai Jiao Tong University Shanghai 200240 P. R. China; Haiyu Chemical Engineering Co. Ltd Dongying 257506 China

## Abstract

Layered two-dimensional (2D) membranes hold great promise in the study of confined ion transport and nanofluidic applications. However, 2D layered membranes suffer from poor stability in water and harsh chemical conditions. Here we use amorphous silica-based nanosheets obtained by vermiculite to assemble a layered glass membrane (LGM) with a 2D nanofluidic channel network in the interlayer space. We find that the water stability and corrosion resistance of the LGM are improved compared to that of the layered vermiculite membrane. Moreover, the surface charge-governed proton conductivity of LGM remains stable at about 4 × 10^−2^ S cm^−1^ when the HCl solution concentration changes by orders of magnitude. The enhanced stability of LGM is of great significance for the study of confined ion transport and is expected to be applied to more nanofluidic applications, such as water treatment, molecular sieving, and osmotic energy conversion.

## Abstract 2

We use amorphous silica-based nanosheets obtained by vermiculite to assemble a layered glass membrane with a 2D nanofluidic channel network in the interlayer space.

## Graphical abstract

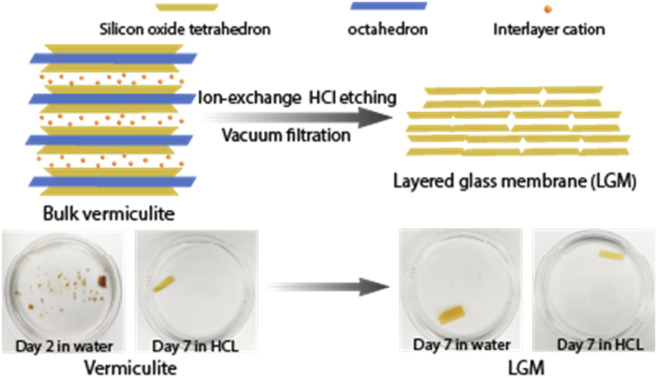

## Introduction

Two-dimensional nanosheets dispersed in solution can be readily reassembled into a 2D lamellar nanostructured membrane, and have been recognized as promising nanofluidic building materials,^1,2^ showing great potential in applications such as proton transport,^3^ water treatment,^4–6^ information processing,^7^ and osmotic energy conversion.^8–10^ Recently, many 2D materials have been used to construct nanofluidic channels.^3,11–13^ However, many of them suffer from poor stability, either in water or in relatively harsh chemical conditions. For example, GO membranes can easily disintegrate in aqueous environments if not crosslinked.^14,15^ Meanwhile, GO is highly prone to reduction which will compromise its hydrophilicity.^16^ Compared with GO, MXene membranes show excellent water stability,^12^ but they can be easily oxidized in the ambient environment.^17–19^ Vermiculite, a natural clay mineral with a sandwich structure consisting of one layer of octahedral sheet and two layers of tetrahedral silicate (Fig. S1†), is highly stable against reduction or oxidization. It is highly economical and nature-occurring material with the global production reaching 0.5 million tons per year.^20^ The exfoliated vermiculite 2D nanosheets can also be assembled into laminar film for proton transport,^3^ oil/water separation,^21^ ion sieving,^22^ lithium-ion battery,^23^ and osmotic power generation.^24^ However, vermiculite membranes have poor stability in water^14^ without the help of crosslinking cations or molecules. In addition, although it was used for proton transport, it is actually unstable in acidic environment because the octahedral sheet of the vermiculite nanosheet is essentially composed of oxide and hydroxide of Al^3+^, Mg^2+^. Therefore, it is still highly demanded to search for novel 2D nanofluidic material that is stable in water and acidic environment.

In this work, we report a 2D layered glass membrane (LGM) with improved aqueous and acid stability. The LGM is derived from vermiculate nanosheet by etching away the octahedral layer. The LGM can keep its structure after several days of immersion in water and acidic solutions.

## Results and discussion

**Fig. 1:**
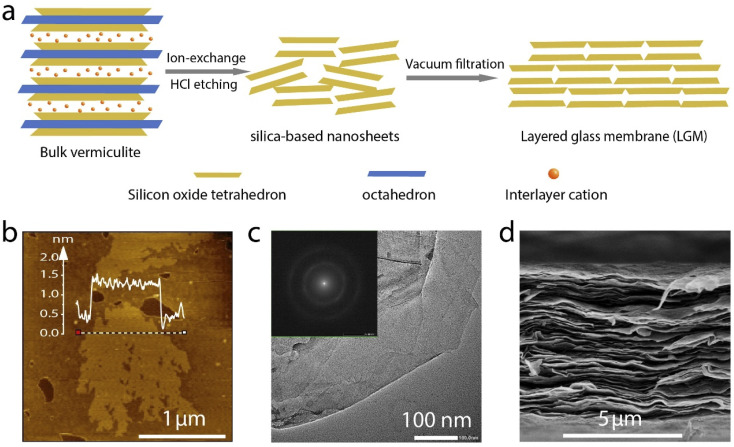
Fabrication and characterization of layered glass membrane. (a) Schematic illustration of the preparation of LGM. (b) AFM (c) TEM and (d) SEM image of the LGM. Bulk vermiculites (left) are exfoliated by ion-exchange and etched to remove the magnesium oxides and aluminum ions into silica-based nanosheets (middle), followed by resembling into a 2D layered glass membrane upon vacuum filtration(right). (b) AFM image of the silica-based nanosheet, revealing its nanosheet structure with a thickness of 1.5 nm. (c) TEM of the silica-based nanosheet, showing its ultrathin nanosheet structural features. Inset is the SAED pattern of the silica-based nanosheet, indicating its amorphous state. (d) Cross-sectional SEM image of the LGM, presenting its ordered laminar structure with a thickness of about 8 μm.

Thermally expanded vermiculite is used in this work to prepare the LGM, as illustrated in Fig. 1a. The vermiculite is first exfoliated into nanosheets by ion exchange as reported in a previous work.^3^ Then, the vermiculite nanosheets are etched with hydrochloric acid to remove the octahedral, yielding glassy silica-based nanosheets. The nanosheets were vacuum filtrated to obtain a layered membrane, termed 2D layered glass membrane (LGM). The presence of hydroxyl groups on the surface of silica-based nanosheets give rise to the negative charges in water, which imparts the membrane with cation selectivity. Atomic force microscopy (AFM) image shows that the nanosheet is partly porous (Fig. 1b), which should be attributed to the etching of the impurities in the vermiculate. Transmission electron microscopy (TEM) measurement was also carried out (Fig. 1c). Selected area electron diffraction pattern (SAED) of the measured region shows no bright and sharp dots, suggesting that LGM are amorphous (the inset of Fig. 1c). In addition, there is no lattice fringes in the TEM images as presented in Fig. S2,† which is in agreement to the result of SAED, again suggesting the amorphous structure of the nanosheet. These indicate that after etching, the nanosheet have undergone phase change, because the original tetrahedral silica sheets of the vermiculate are crystalline. Such phase change is understandable considering that most stable silica materials are amorphous. Based on scanning electron microscopy (SEM) image in Fig. 1d, it can be observed that LGM is laminar, formed by stacked nanosheets, and the thickness of the film is about 8 μm. X-ray diffraction pattern further confirmed the layered structure (Fig. S3†).

Furthermore, we conduct a mapping test on VM and LGM. It can be seen that the silicon-oxygen ratio in LGM increases compared to VM, while the proportion of elements such as aluminum, magnesium, and iron significantly reduced after the acid etching (Tables 1 and S1†). As mentioned above, the mapping results suggest that most of octahedral sheets are etched away, leaving the silica.

**Table 1:** Composition of the layered glass membrane

Element	Mass (%)	Atom (%)
O	48.54	64.37
Si	28.62	21.62
Al	6.96	5.47
Mg	5.10	4.45
Fe	10.79	4.10
Total	100.00	100.00

### Water stability and corrosion resistance

**Fig. 2:**
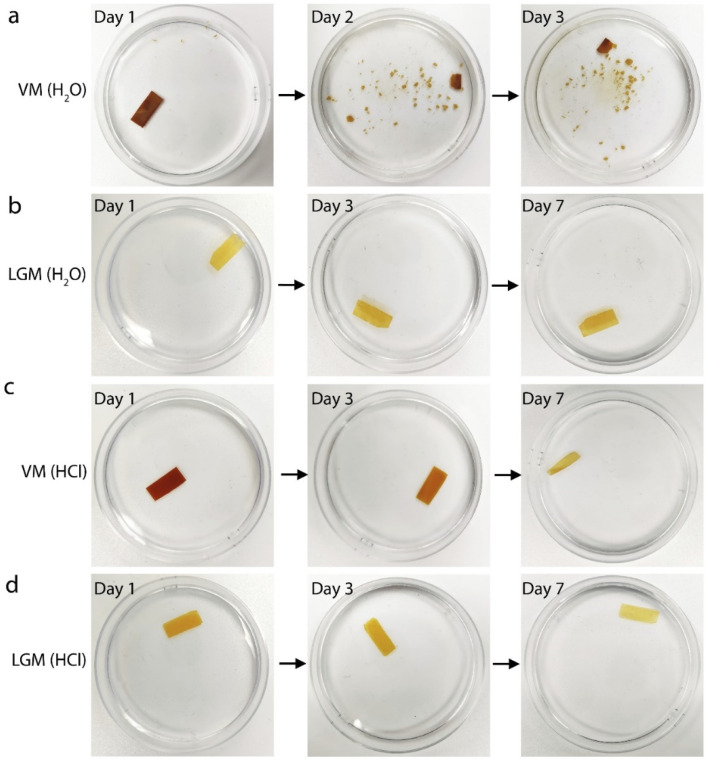
The acid and water stability performance testing. (a and b) are the optical photos of VM and LGM in aqueous solution at different times, respectively. The VM decomposes in water in less than 24 hours. On the contrary, intact LGM remained stable in water after 7 days of immersion. (c and d) are the optical photos of VM and LGM in 1 M HCl solution at different times, respectively. With the increase of immersion time of VM in hydrochloric acid solution, the volume of the VM became smaller, while the size of LGM did not change much after 7 days of immersion in HCl, only the color became lighter.

To verify the water stability and corrosion resistance of LGM, a 12 mm × 5 mm rectangular LGM and a similar sized vermiculite membrane are immersed in ultrapure water and 1 M HCl. By taking the optical images of membranes over time, the water and acid stability of VM and LGM can be intuitively compared. After several days of observation, it can be clearly seen that the VM has disintegrated in the ultrapure after 24 hours (Fig. 2a). In contrast, the structure of the LGM remains stable after 7 days of immersion in ultrapure water (Fig. 2b). This suggests that the reconstructed LGM has higher water stability than VM. It has been proposed that the surface charge density and van der Waals interaction are important factors that influence the water stability of 2D materials.^12^ In our work, the surface of the nanosheets is similar to that of the vermiculite. We speculate that they might have similar surface charge density. Since VM is unstable in water, we further deduce that the surface charge density should not be responsible for the enhanced water stability of the LGM. Therefore, the stability might be attributed to the enhanced van der Waals interaction. However, calculating the van der Waals interaction is challenging for our material because it does not have well defined structure or chemical composition, and demands future studies.

The same stability test is carried out in 1 M HCl solution, as shown in Fig. 2c and d. The VM is gradually corroded by soaking in 1 M HCl solution after 7 days (Fig. 2c). On the seventh day, only a part of VM remained. However, there is no significant change in the structure of the LGM immersed in 1 M HCl solution on the seventh day, revealing that the LGM has better acidic corrosion resistance than the VM. Clearly, this is because most of the octagonal layer has been etched away. The color of the LGM become lighter with increasing immersion time. This is because the residual metal ion impurities are gradually etched away by HCl solution. The observations from these experiments support the findings that the silica-based nanosheet-assembled LGM has higher water stability and corrosion resistance compared to VM, which are important for various nanofluidic applications.

### Proton conductivity

**Fig. 3:**
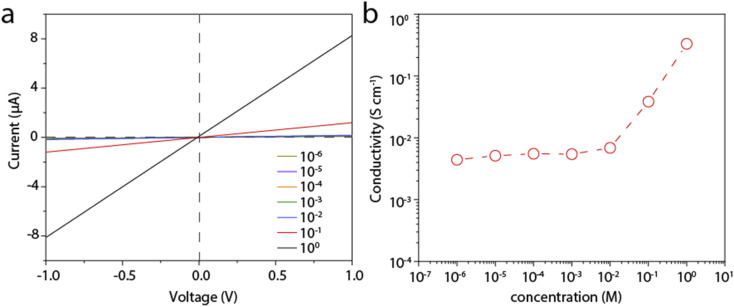
Proton conductivity of the LGM. (a) Typical *I*–*V* curves to test the proton conductivity of the LGM. (b) Calculated proton conductivity of the LGM for different bulk concentration of the HCl, showing a surface-charge-governed transport behavior.

We then measure the proton conductivity using a homemade device by embedding a 12 mm × 5 mm rectangular LGM into a polydimethylsiloxane elastomer (PDMS). Proton transport properties of the LGM are studied by current–voltage (*I*–*V*) measurements, which are performed in HCl aqueous solutions of varying concentrations at room temperature. The typical *I*–*V* curves are shown in Fig. 3a, which exhibit linear ohmic behavior. The conductance in different solutions is calculated based on these curves. It can be seen from the Fig. 3b, when the concentration is higher than 0.01 M, the conductivity value of the solution is proportional to the concentration. When the concentration is lower than 0.01 M, the conductivity value of the solution is largely independent of the concentration of HCl solution. When the concentration changes by four orders of magnitude from 1 μM to 0.01 M, the conductivity keeps at ∼4 × 10^−2^ S cm^−1^. This result reflects a typical surface-charge-governed transport behavior.^25,26^ Due to the negative surface charge of the nanosheets, additional protons are attracted near the nanosheet surface, *i.e.*, inside the channel. As a result, the conductivity is increased. In addition, the phenomenon in Fig. 2c and d also shows that the test process of HCl solution will have a slight effect on LGM, and the lightening of LGM may be accompanied by a decrease in thickness or quality. In the process of calculating the conductivity of the *I*–*V* curve, the decrease in thickness or quality will make the calculation result slightly smaller, and the actual conductivity value measured by the conductivity meter will be a little higher. It can be seen from the above that when the bulk concentration of the HCl is low, the conductivity is governed by these additional protons, or the surface charge, which is independent of the bulk concentration. However, when the bulk concentration is high, the conductivity contributed by these additional protons can be neglected with the bulk conductivity. Consequently, the conductivity is linearly correlated to the bulk concentration. We also tested other electrolytes, including KCl, NaCl, and CaCl_2_, all of which showed similar surface-charge-governed transport behavior (Fig. S4–S6†). In Fig. 3 and S4–S6,† it is shown that the surface of LGM nanosheets has negative charges, which control the internal channel transport behavior. It is worth noting that the negative charges between the layers repel anions and attract cations, which carry most of the ionic current. In the absence of applied voltage, the direction of ionic current is consistent with a net flow of positive charges from high to low concentrations, indicating that this LGM is cation-selective.

**Fig. 4:**
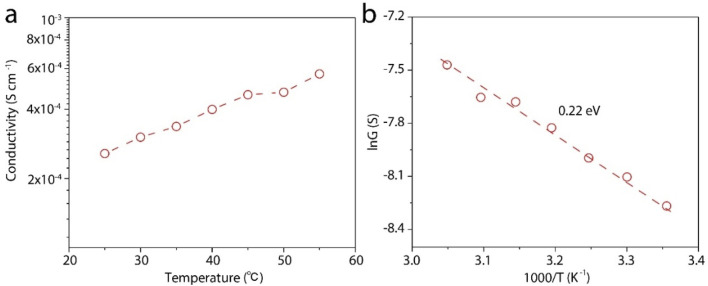
Activation energy of the proton transport. (a) The proton conductivity of LGM at different temperatures in 10^−4^ M HCl solution. (b) Arrhenius plot of proton conductance, showing a low activation energy of 0.22 eV.

To gain deeper insight into the proton transport mechanism, we measured the proton conductivity at different temperatures. Before testing, the device is immersed in the test solution and placed in each temperature environment for half an hour. Fig. 4a shows the proton conductivity of the LGM. We further plot the log scale of the conductivity against 1000/*T*, where *T* is the absolute temperature. In this way, we can calculate the activation energy (*E*_A_) of the proton transport, ln *G* ∝ −*E*_A_/*kT*, where *k* is the Boltzmann constant. Based on this equation, we calculate *E*_A_ to be 0.22 eV. This is a low activation energy, and may suggest the fast Grotthus transport mechanism.^27^

### Stable proton transport in acidic environment

**Fig. 5:**
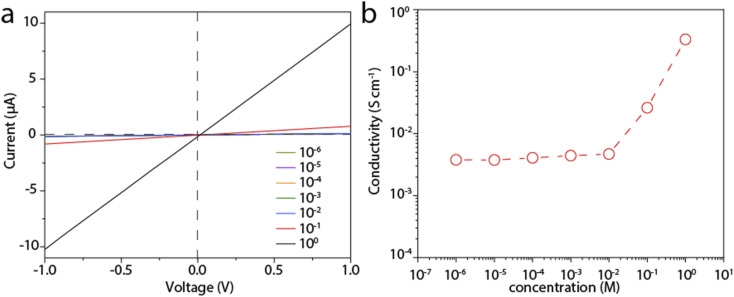
Stability of the proton conductivity of the LGM in acidic environment. We immersed a LGM in 1 M HCl solution for 7 days, and tested the proton conductivity again. (a) Typical *I*–*V* curves. (b) Calculated proton conductivity of the LGM for different bulk concentration of the HCl, showing that the proton conductivity is hardly changed compared to that before immersion.

Fig. 2 shows the improved stability of LGM in acidic environment. We expect that LGM should also show stable proton conductivity in acidic environment. To demonstrate such, we perform the same proton conductivity test as in Fig. 3 using a LGM immersed in HCl solution for 7 days. The proton conductivity still keeps the high value of 4 × 10^−2^ S cm^−1^ when the bulk concentration changes from 1 μM to 0.01 M. These results confirm that the proton conductivity is also highly stable in acidic environment. We note that Fig. 2 suggests that HCl treatment etches away more metal impurities. However, such etching does not necessarily induce the changes in surface charge density (Fig. 5).

## Conclusions

Our work shows that layered glass membrane assembled with glassy silica nanosheets derived from vermiculate is a promising nanofluidic material for proton transport. Compared to layered vermiculate membrane, it has better stability in water and acidic environment. It also has better chemical stability than graphene oxide membrane and MXene membrane. Therefore, we expect it find important applications in a range of nanofluidic applications, such as energy conversion and water treatment.

## Experimental section

### Preparation of vermiculite nanosheet

The vermiculite (40 mesh, Hebei Yanxi, China) used in this experiment was thermally expanded vermiculite. Firstly, the vermiculite was exfoliated into nanosheets. 5 g vermiculite were added to 100 ml of saturated sodium chloride solution, stirred at 110 °C for 24 hours, and then washed with 100 ml deionized water by repeated centrifugation 6–7 times until no chloride ions could be detected in the supernatant with silver nitrate. The products were then mixed with 2 M 100 ml lithium chloride solution, stirred at 110 °C for 24 hours, and then washed with 100 ml deionized water by repeated centrifugation 6–7 times until no chloride ions could be detected in the supernatant with silver nitrate. After that, the product was mixed with 100 ml H_2_O_2_, stirred again at 110 °C for 24 hours. The 6–9 hours of ultrasonication were used to complete the exfoliation. Finally, it was centrifuged to take the supernatant (8000 rpm, 5 min).

### Preparation of layered glass membrane

The obtained vermiculite nanosheet colloidal solution was diluted to 0.5 g ml^−1^. Then 100 ml solution was mixed with 100 ml of 0.6 M HCl solution, and stirred at 60 °C for 6 hours to obtain a shallow yellow colloidal solution. Finally, 600 ml of the above reaction product was vacuum filtered to obtain the layered glass membranes (LGM). During this period, deionized water was repeatedly added to wash the LGM until no chloride ion could be detected in the bottom liquid of suction filtration.

### Preparation of reagents

In the preparation of vermiculite and layered glass membrane, the main drugs we use are: Sodium chloride (NaCl, AR, SCR); Lithium chloride monohydrate (LiCl, MACKLIN); Hydrogen peroxide (H_2_O_2_, AR, 30%, SCR); Hydrochloric acid (HCl, AR, SCR); Absolute ethyl alcohol (C_2_H_5_OH, AR, SCR); Potassium chloride (KCl, AR, MACKLIN); Calcium chlorid (CaCl_2_, AR, MACKLIN) and Dimethyl silicone polymer (PDMS, Dow Corning).

### Material characterization

The LGM were investigated by a Scanning electron microscope (SEM, S-4800), a transmission electron microscopy (TEM, JEM-F200) and an atomic force microscope (AFM, Agilent 5400). Entertainment Maximized (EMAX, S-4800) instrument was used to analyze the content of elements in LGM, The instrument was used with SEM, and the significance of mapping is mainly to understand the regional distribution of material elements. The accelerating voltage of TEM in Fig. 1c is 200 kV, and the accelerating voltage of SEM in Fig. 1d is 5.0 kV. X-ray diffraction (XRD, Smart Lab 9 kW) was used to perform X-ray diffraction of LGM and analyze its diffraction pattern, and the X-ray wavelength used was 0.54 nm.

### Ion conductivity measurement

We measure the proton conductivity using a homemade device by embedding a 12 mm × 5 mm rectangular LGM into a polydimethylsiloxane elastomer (PDMS). After curing, we carved two reservoirs out of the PDMS. The two ends of the LGM were exposed to the reservoirs. Conductivity was measured by a Keithley 2450 device by sweeping the voltage from −1 V to 1 V with a sweep step of 21 steps and a delay time of 0.1 s. Ag/AgCl electrodes were used to apply the voltage.

## Author contributions

G. J. conceived and designed the project. G. J. and Q. L. supervised the project. K. L. conducted the experiments. K. L., T. H., G. J., and Y. T. contributed to data analysis and manuscript drafting. All authors contributed manuscript revision.

## Conflicts of interest

The authors declare no competing financial interest.

## Supplementary Material

RA-012-D2RA03848J-s001
