# Supplementary material for: Nanofluidic proton channels based on a 2D layered glass membrane with improved aqueous and acid stability
Source: RSC Adv. 2022 Oct 17;12(46):29640–6. doi: 10.1039/d2ra03848j (PMC9574643; doi:10.1039/d2ra03848j)
Supplement: RA-012-D2RA03848J-s001 [file RA-012-D2RA03848J-s001.pdf]

Electronic Supplementary Material (ESI) for RSC Advances.
This journal is © The Royal Society of Chemistry 2022

Supporting Information for

Nanofluidic proton channels based on 2D layered glass membrane with improved aqueous and acid stability

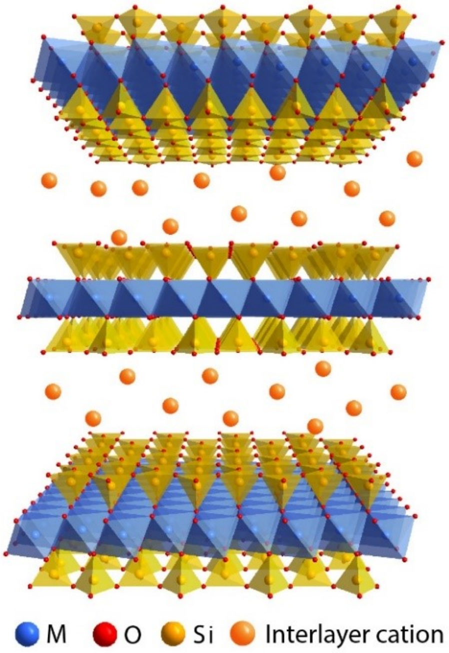

Figure S1 The sandwich structure of vermiculite. The yellow layers are silicon-oxygen tetrahedron, and the blue layer is magnesium/aluminum-based octahedron. M stands for metal cation impurities such as Al^{3+}, Mg^{2+}, Fe^{3+}.

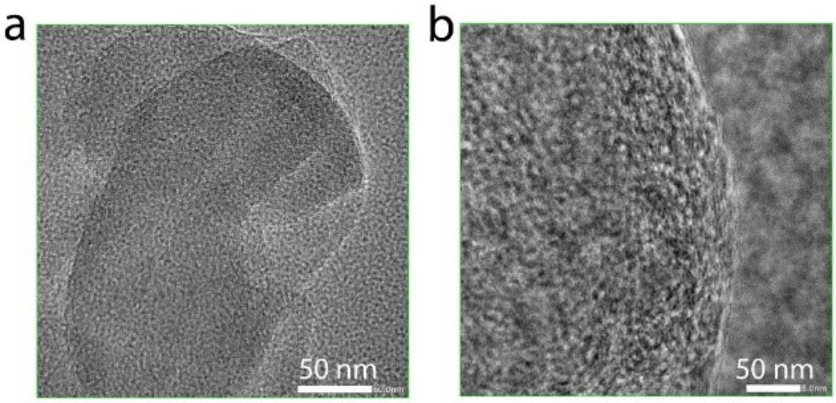

Figure S2 TEM of 2D silica nanosheets. The 2D silica nanosheets nanosheet has no obvious lattice fringes, indicating that it is amorphous.

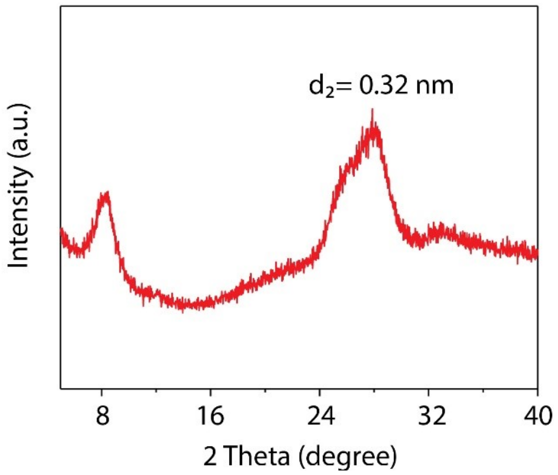

Figure S3 X-ray diffraction image of LGM. The broad band between 24° to 30° is assigned to amorphous silica. The peak at 8.2° corresponds to the interlayer spacing, and suggests the laminar structure.

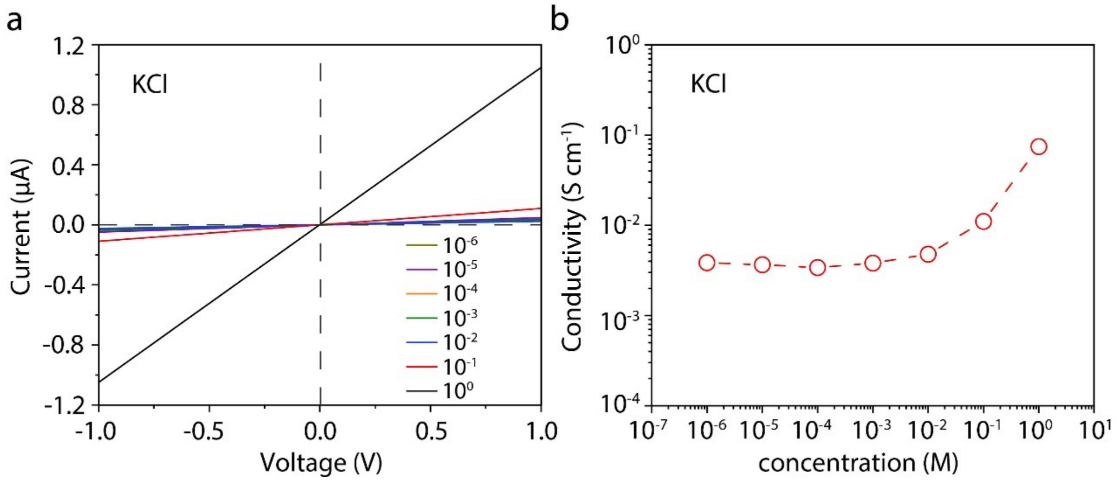

Figure S4 (a) *I-V* characteristic curve under concentrations of KCl solution. (b) The variation of the conductivity of LGM nanochannels under varying concentrations of KCl solution.

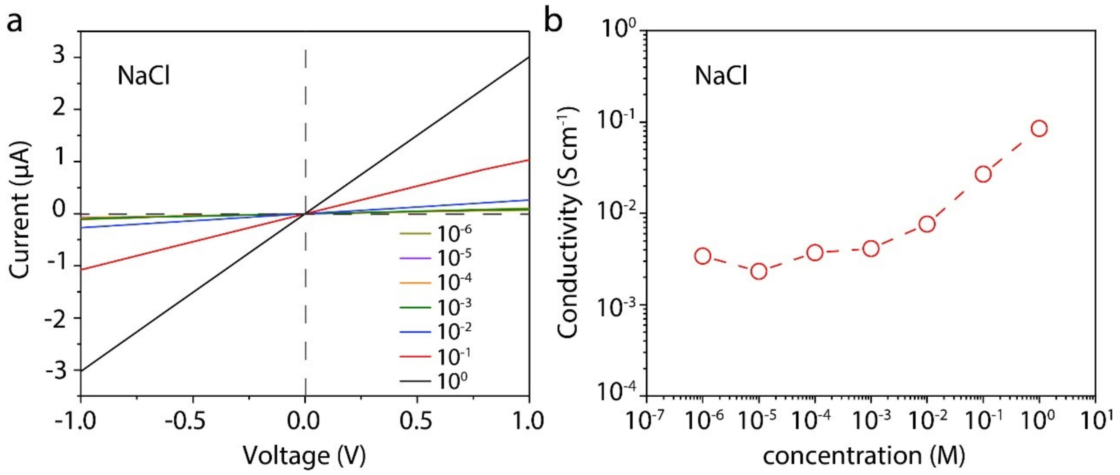

Figure S5 (a) I-V characteristic curve under concentrations of NaCl solution. (b) The variation of the conductivity of LGM nanochannels under varying concentrations of NaCl solution.

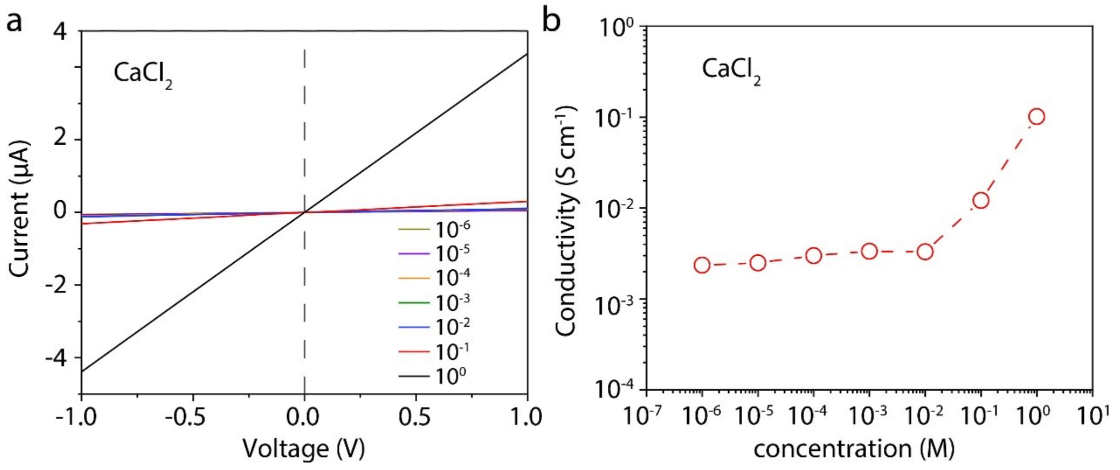

Figure S6 (a) I-V characteristic curve under concentrations of $CaCl_2$ solution. (b) The variation of the conductivity of LGM nanochannels under varying concentrations of $CaCl_2$ solution.

Table S1 Element composition of layered vermiculite membranes (VM)

Element	Mass (%)	Atom (%)
O	55.56	68.41
Si	18.48	12.96
Mg	13.99	11.34
Al	8.12	5.93
Fe	3.85	1.36
Total	100.00	100.00
